# Association of the Co-stimulatory Molecules Polymorphisms with CMV Infection in Liver Transplant Recipients

**Published:** 2011-11-01

**Authors:** M. H. Karimi, M. Motazedian, B. Geramizadeh, S. Nikeghbalian, R. Yaghobi, F. Abedi, M. Hossin Aghdaee, N. Azarpira, M. Arabpour, Z. Malekpour, M. Namayandeh

**Affiliations:** 1 *Transplant Research Center, Shiraz University of Medical Sciences, Shiraz, Iran*; 2 *Islamic Azad University, Jahrom branch, Jahrom, Iran*

**Keywords:** CMV, Transplant recipient, polymorphisms, Liver, Rejection, pathogenesis

## Abstract

Background: Co-stimulatory molecules play a critical role in regulating T-cell function during CMV infection after liver transplantation.

Objective: To investigate the relationship between the polymorphisms of the co-stimulatory genes and the susceptibility to CMV infection after liver transplantation.

Methods: Single nucleotide polymorphisms (SNPs) in PD-1 gene (PD1.1 A/G, PD1.3 A/G, PD1.9 C/T) ICOS (-693 A/G, 1720 C/T), CTLA-4 gene (318 C/T, 1722 T/C, 1661 A/G, 49 A/G) and CD28 (+17 C/T) were analyzed by PCR-RFLP in 70 liver transplant patients. CMV infection was determined in these patients by antigenemia test.

Results: CTLA-4 49G showed significant association with CMV infection (p=0.03, OR=3.82, 95% CI: 0–3.5; p=0.01, OR=004, 95% CI: 0–1.3). G and T alleles in CTLA-4 gene (318 C/T and 1661 A/G) (p=0.03, OR=0, 95% CI: 0–3.5; p=0.01, OR=0.04, 95% CI: 0–1.3) were significantly higher in CMV-infected rejector group.

Conclusion: CTLA-4 have significant role in CMV pathogenesis and rejection among CMV-positive liver transplant patients.

## INTRODUCTION

Early infection of patients with liver transplant may be problematic. Because of compromised immunity, transplant recipients are at risk of developing various infectious diseases especially cytomegalovirus (CMV). CMV is the most common viral pathogen of allograft rejection in liver transplant recipients. CMV disease in patients after liver transplantation is not only usually fatal, but remains a principal reason of morbidity and increased cost post-transplantation [1]. In addition to its direct diverse effects, it has also several indirect impacts, including increased allograft rejection rate [2].

A number of host genetic factors have single nucleotide polymorphisms (SNPs) in different genes. For example, cytotoxic T-lymphocyte associated protein 4 (CTLA-4) gene polymorphisms have serious effects on a variety of clinical courses accompanied by hepatitis B virus (HBV) and CMV infection [3-5].

There are several polymorphisms in co-stimulatory molecules PD-1, CD28, CTLA-4 and ICOS. All of these molecules are on the T lymphocyte surface and take part in the immune response regulation [6].

In the last few years, several genetic SNPs in CTLA-4 and its ligand have been recognized and demonstrated to influence the intensity of T cell-mediated immunity, resulting in various autoimmune diseases, and even the occurrence of post-transplant allograft rejection [7, 8].

Both CTLA-4 and CD28, as co-stimulatory molecules on T-cells, are bound to the same B7 ligand on antigen-presenting cells (APCs). CD28 molecule is expressed on T-cells and induces T-cell responses. CTLA-4 is expressed on the activated T cells and prevents T-cell activation by weakening CD28/B7 interactions through its higher affinity for B7 [9].

CTLA-4/CD28 gene polymorphisms are genetically associated with several autoimmune diseases such as systemic lupus erythematosus (SLE), Graves’ disease, rheumatoid arthritis and Behçet’s disease [10, 11]. Moreover, CTLA-4/CD28 gene polymorphisms have been shown to affect the inhibitory/activating functions of CTLA-4 and CD28, respectively [12].

The programmed cell death-1 (PDCD1, also known as PD-1) gene is one of the co-stimulatory molecule genes located on chromosome 2q37.3 which encodes a cell surface receptor named PD-1 [13]. This inhibitory immunoreceptor is expressed on activated T cells, B cells, and myeloid cells [14]. PD-1 has a principal assignment in T cell regulation during HBV and CMV infection. It is considered that PD-1 is one of the genes that increase susceptibility to HBV infection and disease progression [15].

Considering the great effect of co-stimulatory molecules on T-cell function and assuming that alteration of gene polymorphisms expression or function would possibly influence the immune response, this study was conducted to investigate the probable connection between 10 polymorphisms of co-stimulatory molecules (four polymorphisms in CTLA-4, three polymorphisms in PD-1, two polymorphisms in ICOS, and one polymorphism in CD28) with HBV and CMV infection in liver transplant patients.

## MATERIALS AND METHODS

Patients 

The study group composed of 70 patients (27 women and 43 men) aged between 2 and 62 years, who received a liver from donors since 2007 to 2010 in the Transplantation Center of Nemazi Hospital, Shiraz University of Medical Sciences, Shiraz, southern Iran. The prevalence of CMV infection for each patient was evaluated by antigenemia test.

DNA extraction

The buffy coat of whole blood taken from liver transplant patients was available in the sample bank of Transplant Research Center. Genomic DNA was extracted from buffy coat, using a QIAamp DNA MiniKit (Qiagen, Germany) according to the manufacturer’s instructions.

Genotyping

We selected four SNPs from the CTLA-4 gene (318 C/T, 1722 T/C, 1661 A/G, +49 A/G), three SNPs from PD.1 gene (PD-1.1 A/G, PD-1.3 A/G, PD-1.9 C/T), two SNPs from ICOS gene (693 A/G, 1720 C/T) and one SNP from CD28 (IVS3+17 C/T) gene in chromosome 2q33-37.

Co-stimulatory molecules gene polymorphisms were evaluated by polymerase chain reaction (PCR)-RFLP.

PCR conditions:

PCR cycles and primers are summarized in Tables 1 and 2. PCR-RFLP method was carried out for PD1.1 A/G, PD1.3 A/G, PD1.9 C/T [16, 17], CD28 +17 C/T [18], and CTLA-4 1722 T/C, 1661 A/G, 318 C/T, +49 A/G [19, 20] and ICOS 693 A/G, +1720 C/T genotyped in 10 µL reaction mixture [21]. After PCR, the products were digested by restriction enzyme and the amplified products were monitored by agarose gel electrophoresis and ethidium bromide staining.

**Table 1:** The Primers and types of PCR used for genotyping of the ICOS, CD28, PD-1 and CTLA-4

Locus	Primer	Method
ICOS 1720 C/T	Forward primer: 5´- TTACCAAGACTTTAGATGCTTTCTT-3´ Reverse primer:5´-GAATCTTTCTAGCCAAATCATATTC-3´	NcoI-based RFLP
ICOS 693 A/G	Forward primer:5´-ATTTCTATCTTATGCTAGGTGCTCCA-3´ Reverse primer:5´-ATCTTGGAAGCGCGTTCAGACTT-3´	BseGI-based RFLP
CD28 +17 C/T	Forward primer:5´-TTTTCTGGGTAAGAGAAGCAGCGC-3´ Reverse primer :5´-GAACCTACTCAAGCATGGGG-3´	Eco47III-based RFLP
PD1.1 A/G	Forward primer: 5´-CTCAACCCCACTCCCATTCT-3´ Reverse primer:5´-TTCTAGCCTCGCTTCGGTTA-3´	MspI-based RFLP
PD-1.9 7625 T/C	Forward primer:5´- GGACAGCTCAGGGTAAGCAG-3´ Reverse primer:5´- AGGGTCTGCAGAACACTGGT-3´	Bpu10I-based RFLP
PD-1.3 7146 A/G	Forward primer: 5-CCCCAGGCAGCAACCTCAAT-3 Reverse primer: 5-GACCGCAGGCAGGCACATAT-3	PstI-based RFLP
CTLA4-1722T/C	Forward primer:5´-CTAAGAGCATCCGCTTGCACCT-3´ Reverse primer:5´- TTGGTGTGATGCACAGAAGCCTTTT-3´	BbvI-based RFLP
CTLA-4 1661 A/G	Forward primer:5´- CTAAGAGCATCCGCTTGCACCT-3´ Reverse primer:5´- TTGGTGTGATGCACAGAAGCCTTTT-3´	MseI-based RFLP
CTLA4 +A9 A/G	Forward primer:5´- GCTCTACTTCCTGAAGACCT-3´ Reverse primer:5´- AGTCTCACTCACCTTTGCAG-3´	BbvI-based RFLP
CTLA-4 318 T/C	Forward primer:5´- AAATGAATTGGACTGGATGG-3´ Reverse primer:5´- TTACGAGAAAGGAAGCCGTG-3	MseI-based RFLP

**Table 2:** The PCR Program used for genotyping the ICOS, CD28, PD-1 and CTLA-4

Locus	PCR Condition
ICOS 1720C/T	1 cycle: 95 °C 5 min; 35 cycles: 95 °C 50 sec, 59 °C 60 sec, 72 °C 60 sec; 1 cycle: 72 °C 7 min
ICOS 693 A/G	1 cycle: 95 °C 5 min; 30 cycles: 95 °C 50 sec, 61 °C 60 sec, 72 °C 60 sec; 1 cycle: 72 °C 7 min
CD28 +17 C/T	1 cycle: 94 °C 5 min; 35 cycles: 94 °C 30 sec, 58 °C 30 sec, 72 °C 30 sec; 1 cycle: 72 °C 7 min
PD1.1 A/G	1 cycle: 95 °C 4 min; 30 cycles: 95 °C 60 sec, 59 °C 60 sec, 72 °C 60 sec; 1 cycle: 72 °C 5 min
PD-1.9 7625 T/C	1 cycle: 95 °C 4 min; 30 cycles: 94 °C 60 sec, 57 °C 60 sec, 72 °C 60 sec; 1 cycle: 72 °C 5 min
PD-1.3 7146 A/G	1 cycle: 95 °C 5 min; 30 cycles: 95 °C 60 sec, 62 °C 60 sec, 72 °C 60 sec; 1 cycle: 72 °C 5 min
CTLA-4 1722 T/C	1 cycle: 95 °C 5 min; 30 cycles: 95 °C 60 sec, 58 °C 60 sec, 72 °C 60 sec; 1 cycle: 72 °C 5 min
CTLA-4 1661 A/G	1 cycle: 94 °C 5 min; 30 cycles: 94 °C 60 sec, 58 °C 60 sec, 72 °C 60 sec; 1 cycle: 72 °C 5 min
CTLA-4 +A9 A/G	1 cycle: 95 °C 4 min; 30 cycles: 95 °C 60 sec, 55.5 °C 60 sec, 72 °C 60 sec; 1 cycle: 72 °C 5 min
CTLA-4 318 T/C	1 cycle: 95 °C 5 min; 30 cycles: 95 °C 60 sec, 58 °C 60 sec, 72 °C 60 sec; 1 cycle: 72 °C 5 min

Antigenemia

The prevalence of active CMV infection was evaluated for each patient by antigenemia test (CMV Brite Turbo Kit, Holland) according to the manufacturer’s instructions.

Statistical analysis 

Allele and genotype frequencies were calculated in patient and control subjects by direct gene counting. Statistical evaluation was carried out using the SPSS ver 15. The frequencies of alleles/genotypes were compared in cases and controls by χ^2^ test and Fisher’s exact test where appropriate. Odds ratios and 95% confidence intervals (CIs) for relative risks (RRs) were calculated. A two-tailed p<0.05 was considered statistically significant.

## RESULTS

Among 70 consecutive recipients, 43 male and 27 female with a mean±SD age of 28.73±14.03 years were studied. The mean±SD age of patients who had an acute rejection episode was 25.77±13.11 years; in patients with normal organ function, it was 31.69±14.96 years (p=0.415). The acute rejection rate in this group was 31%.

Genotype frequencies of CTLA-4 (318 C/T, 1661 A/G) variants were significantly different between rejectors and non-rejectors in CMV-infected patients. In the rejector group patients, for CTLA-4 (318 C/T, 1661 A/G) CC and AA genotypes were significantly more prevalent (p=0.03, OR=0, 95% CI: 0–3.5; p=0.01, OR=0.04, 95% CI: 0–1.3). CTLA-4 (318C/T, 1661 A/G) C and A alleles were significantly more frequent than T and G alleles (p=0.03, OR=0, 95% CI: 0–3.5; p=0.01, OR=0.04, 95% CI: 0–1.3) in the rejector group patients.

**Table 3:** Results of CTLA-4 (318 C/T, 49 A/G, 1722 C/T, 1661 A/G), PD-1 (PD1.1 A/G, PD1.3 A/G, PD1.9 C/T), ICOS ( 693 A/G, +1720 C/T), CD28 (+17 C/T) single nucleotide polymorphism determined in 70 liver transplant subjects with or without CMV infection. In genotypes, each p value is the result of comparing corresponding row with the sum of other rows’

Gene	Genotype	CMV+%(N)	CMV-%(N)	p-value	OR	95%CI
49AG	AA	66.6%(12)	82.6%(43)	0.1	0.42	0.11-1.66
	AG	33.3%(6)	11.53%(6)	0.03*	3.83	0.8-16.9
	GG	0% (0)	5.7%(3)	0.2**	0	0-6.81
	A	83.3%(30)	88.4%(92)	0.4	0.65	0.2-2.15
	G	16.6%(6)	11.53%(12)			

n: Absolute number; CI: Confidence interval; OR: Odds ratio;

* Considered statistically significant with p value threshold of 0.05.

** Considered statistically significant after the Bonferroni correction (p value threshold of 0.017).

Genotypes frequencies of CTLA-4 (+49 A/G) variants were significantly different between CMV-positive and CMV-negative patients. For CTLA-4 (+49 A/G) AA genotype was significantly more frequent (p=0.03, OR=3.82, 95% CI: 0-3.5; p=0.01, OR=0.04, 95% CI: 0–1.3).

**Table 4:** Results of CTLA -4 (318C/T, 49A/G, 1722C/T, 1661A/G), PD-1 (PD1.1 A/G, PD1.3 A/G, PD1.9 C/T), ICOS (-693 A/G, +1720 C/T) CD28 (+17 C/T) single nucleotide polymorphism determined in CMV infected liver transplanted subjects

Gene	Genotype	Reject%(n)	Non-Reject%(n)	P-value	OR	95%CI
318C/T	CC	66.6% (2)	100% (15)	0.03*	0	0-3.56
	CT	33.3% (1)	0% (0)	0.03*		
	TT	0% (0)	0% (0)			
	C	83.3% (5)	100% (30)	0.03*	0	0-3.51
	T	16.6% (1)	0% (0)			
1661A/G	AA	33.3% (1)	93.3% (14)	0.01**	0.04	0-1.30
	AG	66.6% (2)	6.66% (1)	0.01**	28	0.7-5428
	GG	0% (0)	0%(0)			
	A	66.6% (4)	96.6% (29)	0.01**	0.07	0-1.35
	G	33.3% (2)	3.3% (1)			

n, absolute number; CI, Confidence Interval; OR, Odds ratio;

* Statistically significant;

** Considered significant after the Bonferroni correction (P value threshold of 0.017).

## DISCUSSION

In the present study, we studied 10 polymorphisms in patients with liver transplant of PD-1 gene (PD1.1 A/G, PD1.3 A/G, PD1.9 C/T), ICOS (693 A/G, 1720 C/T), CTLA-4 gene (318 C/T, 1722 T/C, 1661 A/G, 49 A/G), and CD28 (IVS3+17 C/T) and their association with CMV infection.

The results showed that genotype frequencies of CTLA-4 (318 C/T, 1661 A/G) variants were significantly different between rejectors and non-rejectors in CMV-positive patients. For example, at CTLA-4 (318 C/T, 1661 A/G) SNP locus, the rejector group had lower frequencies of CT and AG genotypes and C and G alleles. Also, genotypes frequencies of CTLA-4 (+49 A/G) variants were significantly different between CMV-positive and CMV-negative patients. 

Since the functions of CD28, CTLA-4 and ICOS are related to the regulation of T-cell responses, the genotypic co-association of these genes should also be studied. Apart from one polymorphism in leader peptide of CTLA-4, no variation had been found in amino acid sequence of ICOS, CTLA-4 or CD28 molecules. Thus, the genetic linkage between chromosome region 2q33 and autoimmune diseases might not arise from common structural variants of the above molecules, and may demonstrate a role of non-coding polymorphisms [22].

A promoter region is the transcription factor binding site regulating the level of gene expression. There are several reports describing that gene expression can be regulated by variation in non-coding regions [23]. Variations of introns can influence the pattern of mRNA alternative splicing. For instance, splicing variants of CTLA-4 and CD28, coding for putative soluble isoforms of these molecules have been observed in resting T cells [24]. CTLA-4 has distinct independent effects during different phases of T cell responses, such as threshold setting for T cell activation, suppression of T cell proliferation, and apoptosis induction in activated T cells. Since B cells express after cell-cell contact with activated T cells, it is assumed that CTLA-4 can even assisting the regulation of B cell responses directly [25, 26]. The gene polymorphism +49 G/A is the most widely studied one among all the CTLA-4 gene variants, since this is the only polymorphism in the CTLA-4 gene that can change an amino acid [27]. Also, CTLA-4 +49 G allele has also been correlated with incomplete glycosylation of the signal peptide and changed processing in the endoplasmic reticulum, which influences the CTLA-4-driven down-regulation of T-cell activation and is of great value in the pathogenesis of autoimmune diseases [28].

Previous studies proved the association between CTLA-4 and disease occurrence. For instance, the presence of susceptibility alleles at the 1722, 1661, and 318 promoter positions of CTLA-4 did not demonstrate a significant relationship with a graft versus host disease (GVHD) incidence, or disease recurrence [29]. CTLA-4 1661 G and 318 T allele carrier’s frequencies were significantly higher in breast cancer patients than in the control group [30]. Nevertheless, our results did not compatible with the above results. It had been demonstrated that SNPs in CTLA-4 gene are implicated in the susceptibility to HBV infection [11–13], suggesting PD-1 and CTLA-4 SNPs interactions for discovering the disease diversity and clinical course of HBV infection. Hence, further investigation is needed to explain the role of coordination of PD-1 and CTLA-4 polymorphisms in HBV infection. 

This study has several limitations which may prevent the interpretation of our results, including the relatively small number of patients studied, the lack of functional elucidation of the co-stimulatory SNP, and the ethnic differences of SNPs [30]. Our results explanation requires further study in a large population to clarify its significance.

In conclusion, among 10 polymorphisms in CD28, CTLA4, ICOS, and PD-1 region, we found that CTLA-4 49 G has significant association with CMV infection. Also, G and T alleles in CTLA-4 gene (318 C/T and 1661 A/G) are significantly detected in CMV-infected rejector group patients.
